# Genomics in Equine MEED: Whole-Genome Sequencing and Target Mutation Identification

**DOI:** 10.3390/ani16101560

**Published:** 2026-05-21

**Authors:** Kayden Tanner, Marshall Mays, Thu Annelise Nguyen, Tomas Lugo

**Affiliations:** School of Veterinary Medicine, Texas Tech University, Amarillo, TX 79106, USA; kaytanne@ttu.edu (K.T.); marshall.mays@ttu.edu (M.M.); annelise.nguyen@ttu.edu (T.A.N.)

**Keywords:** whole-genome sequencing, equine genomics, MEED, rare variants, variant prioritization, gene ontology

## Abstract

Multisystemic eosinophilic epitheliotropic disease (MEED) is a rare and severe equine disorder characterized by chronic eosinophilic inflammation, epithelial disruption, and multi-organ involvement, with an undefined genetic basis. We performed the high-depth (~40×) whole-genome sequencing of an affected horse and compared it to 40 control genomes. Over 6.3 million variants were identified, with moderate- and high-impact variants enriched in low-frequency categories, including rare and private variants absent from the controls. The affected horse was dominated by missense mutations, with relatively few high-impact variants, consistent with the distributed protein-altering effects rather than a single highly penetrant mutation. Gene prioritization and pathway analyses highlighted the disruption of cytoskeletal organization, microtubule dynamics, epithelial integrity, and immune regulation. The network analysis further revealed the interconnected structural and inflammatory pathways, suggesting a link between an impaired epithelial barrier function and immune homeostasis. Together, these findings provide the first population genomic insight into MEED and support a model in which cumulative mutations contribute to the epithelial instability and persistent inflammation characteristic of the disease.

## Simple Summary

Multisystemic eosinophilic epitheliotropic disease (MEED) is a rare equine disorder first described in 1986, and, since then, a growing number of cases have been recognized. The disease is characterized by a severe, progressive pathology and is often associated with a poor prognosis, with affected horses typically euthanized within one year of diagnosis. Clinically, MEED presents with signs including chronic weight loss, diarrhea, cutaneous lesions, and the eosinophilic infiltration of multiple epithelial tissues, reflecting its multisystemic nature. With the recent advances in genomic technologies, we performed whole-genome sequencing on a horse diagnosed with MEED to investigate the potential genetic contributors to disease pathogenesis. This analysis revealed a collection of rare and potentially pathogenic variants that may be associated with disease development. A further evaluation of these variants enabled the identification of biological pathways and systems likely impacted, providing insight into the potential underlying cellular mechanisms of MEED. This study establishes a foundational framework for understanding its genetic basis and guiding future investigations into disease mechanisms and therapeutic strategies.

## 1. Introduction

Multisystemic epitheliotropic disease (MEED) is a rare, progressive inflammatory disorder of primarily young horses, characterized by the widespread eosinophilic infiltration of epithelial tissues across many organ systems [1,2,3,4,5,6,7,8,9,10,11]. It is also commonly referred to as eosinophilic gastroenteritis, eosinophilic enterocolitis, eosinophilic granulomatosis, hypereosinophilia syndrome, and exfoliative eosinophilic dermatitis, and is thought of as a form of the inflammatory bowel disease (IBD) complex [8]. Clinically, affected horses often present with chronic weight loss, diarrhea, pruritus, generalized dermatitis, ventral edema, respiratory signs, and hypoalbuminemia, reflecting the multisystemic nature of this disease [4,8,9,10,12,13,14]. Similarly, epithelial degeneration, fibrosis, and granulomatous inflammation are often seen, suggesting immune-mediated tissue injury [8]. Although the histopathologic features are consistently described, the molecular and genetic determinants of MEED remain undefined. Due to the rarity of reported cases, with only around 50 cases in the past, most of the existing literature has focused on the clinical and pathological characterization, with limited to no exploration of the genomic contributors [1,2,3,4,5,6].

Over the past decade, advances in equine genomics have transformed our understanding of genetic diversity, breed structure, and evolutionary selection in the domestic horse (*Equus caballus*). Large-scale sequencing efforts have revealed a substantial variation across breeds, shaped by domestication, selective breeding, and population bottlenecks [15]. These processes have enriched certain alleles within lineages while reducing the overall genetic diversity in others, influencing disease susceptibility and immune-related traits. In this context, the interpretation of the genomic variation requires a careful comparison against population-level data to distinguish common breed-associated polymorphisms from potentially pathogenic rare and private variants.

In a previous publication, Isolation and Characterization of Equine Lymph Node Endothelial Cells, we established the first primary-lymph-node-derived endothelial cell culture (ELAT) from a horse diagnosed with MEED [16]. That work addressed a significant gap in the equine biomedical research by generating a physiologically relevant associated cell model and included preliminary whole-genome sequencing to confirm the species origin and identify the general variation relative to the EquCab3.0 reference genome. However, that sequencing was exploratory and did not incorporate a comprehensive population-based filtering or rare variant prioritization.

The goal of the present study was to perform high-depth whole-genome sequencing (WGS) at 40× coverage, substantially exceeding the previous 1.87× coverage, and to evaluate the affected genome within a defined population framework. Coverage above the 30× standard for comprehensive variant discovery enables the confident detection of single-nucleotide variants and small insertions and deletions, the accurate discrimination between heterozygous and homozygous genotypes, and a reduction in false positives associated with a sequencing error [17]. The resulting variant dataset was systematically compared to a genomic reference databank of 185 horses, allowing the identification of unique variants to the broader equine population through frequency filtering [18]. This population comparative framework is particularly critical in veterinary genomics, where rare diseases often present as isolated cases and traditional cohort-based genetic association studies are not feasible. By situating an individual genome within the context of population diversity, it becomes possible to systematically filter background polymorphism and prioritize variants that may contribute to immune dysregulation. Moreover, characterizing rare genomic variation in affected individuals contributes to the broader understanding of the equine genetic architecture and enriches the available variant catalogs.

By integrating high-depth whole-genome sequencing with a population-level allele frequency comparison, this study advances the application of genomic investigation into rare equine disease research. Beyond its relevance to MEED, this work contributes to the ongoing efforts in equine genetics to map the variation across individuals and breeds, understand the evolutionary constraints on immune-related genes, and refine the interpretation frameworks for the rare variant discovery. Such approaches are essential for bridging individual disease genomes with the population-scale equine genetic diversity and for expanding the translational potential of equine genomic research.

## 2. Materials and Methods

### 2.1. Cells and Cell Culture

Equine lymph node endothelial cells (ELAT) were maintained and isolated, and whole-genome sequencing (WGS) was conducted according to the protocol described in our previous paper Isolation and Characterization of Equine Lymph Node Endothelial Cells [16]. ELAT cells were used at passage 2 and had not undergone transformation prior to whole-genome sequencing. At this early passage, non-transformed cells were selected to minimize the likelihood of culture-acquired genetic alterations and to better preserve the genomic profile of the original sample.

Genomic DNA was extracted from cultured cells using the QIAamp DNA Micro Kit (QIAGEN, Hilden, Germany) according to a user-developed protocol. DNA purity was evaluated using an Invitrogen™ NanoDrop™ One Spectrophotometer (Thermo Fisher Scientific, Waltham, MA, USA).

### 2.2. Whole-Genome Sequencing Data Processing and Variant Discovery Workflow

Genomic DNA samples were prepared according to Novogene America’s submission guidelines. Libraries were generated using the Abclonal Rapid Plus DNA Library Preparation Kit for Illumina® (ABclonal, Inc., Woburn, MA, USA) and paired-end sequencing (150 bp) was performed on the Illumina NovaSeq X Plus (Illumina, Inc., San Diego, CA, USA) platform. Sequencing achieved a quality threshold of Q30 and generated approximately 90 Gb of raw data. 

Raw sequencing reads were processed in a Linux-based computational environment. Quality control was performed using FastQC (v0.11.9). Reads were aligned to the UCSC EquCab3.0 reference genome (NCBI accession GCF_002863925.1) using BWA-MEM, with a custom reference index generated from the UCSC FASTA. SAM files were converted to BAM format, sorted, and indexed using SAMtools. Duplicate reads were identified and marked using Picard MarkDuplicates (Broad Institute, Cambridge, MA, USA).

To enable comparative analysis, whole-genome sequencing data from 40 control horses were incorporated from a publicly available dataset (“Analyses of whole-genome sequences from 185 North American Thoroughbred horses, spanning 5 generations”; BioProject PRJNA993255) [18]. The cohort was limited to 40 publicly available healthy Thoroughbred horses due to resource and computational constraints. Although the affected individual was reported by the owner as a Quarter Horse, genetic evaluation indicated that the MEED case clustered closely with the Thoroughbred control cohort, supporting its inclusion in this comparative framework (Supplementary S6). Therefore, these controls were used as an available population background to prioritize private or rare variants in the affected sample. We acknowledge that a larger, identically breed-matched control cohort would be preferred for population filtering. This data was included to facilitate identification of variants enriched in the diseased sample relative to a population background.

### 2.3. Structural Variant (SV) and Copy Number Variant (CNV) Detection

Genome-wide copy number variation was assessed using CNVkit v0.9.10. Duplicate-marked, coordinate-sorted, and indexed BAM files aligned to the USC EquCab3.0 reference genome were used as input. Prior to CNV analysis, alignment files were checked for mapping quality, sequencing depth, and genome-wide coverage consistency to ensure that read depth variation reflected biological copy number differences rather than poor alignment or sequencing artifacts.

CNVkit was run using the batch command with the whole-genome sequencing option, which allows depth binning across the entire genome rather than restricting analysis to targeted regions. The EquCab3.0 reference genome was used for bin generation and read depth normalization. An average bin size of 50 kb was selected to balance genomic resolution with signal stability. Smaller bin sizes, including 25 kb, were initially evaluated but produced increased noise, particularly in regions with low mappability or repetitive sequence content. Therefore, 50 kb bins were used for the final analysis to improve confidence in segment-level copy number interpretation.

Read depth was calculated across genomic bins and normalized by CNVkit to account for GC content bias, local sequencing variability, and genome-wide coverage differences. Copy number values were reported as log2 copy ratio estimates, where values near 0 indicate a diploid copy number state, positive values indicate relative copy number gain, and negative values indicate relative copy number loss. 

Segment-level CNVs were generated from normalized bin-level values using circular binary segmentation. Segmented CNV calls were exported as cns files and used to identify larger regions of consistent copy number change across the genome. To reduce false-positive calls, CNV segments were filtered based on both magnitude and size. Only segments with an absolute log2 copy ratio of ≥0.3 and a minimum segment size of ≥50 kb were retained for downstream interpretation. Regions overlapping highly repetitive, poorly mappable, or otherwise ambiguous genomic intervals were interpreted cautiously and removed when applicable.

Both bin-level (.cnr) and segment-level (.cns) outputs were used for downstream visualization and interpretation. Bin-level data were used to show genome-wide read depth variation, while segment-level data were used to summarize copy number gains and losses. Chromosome-level CNV patterns were then evaluated to determine whether large-scale chromosomal gains, losses, or focal copy number alterations were present.

Structural variants were identified using a multicaller approach with Manta v1.6.0 and DELLY. To reduce noise from unplaced scaffolds, analysis was restricted to the primary chromosomes chr1–chr31 and chrX. A primary chromosome BED file was generated from the reference index and used to subset the BAM file before SV calling.

Manta was run in germline mode using the primary chromosome BAM file, EquCab3.0 reference genome, and the primary chromosome BED file as the call Regions input. DELLY was run independently for each SV class, including deletions, duplications, inversions, insertions, and breakend events. DELLY calls were then filtered using the germline filtering option and merged into a combined VCF.

Raw SVs were filtered to retain high-confidence events. For intrachromosomal SVs, variants were required to be located on chr1–chr31 or chrX, have defined start and end coordinates, be ≥500 bp in size, have QUAL ≥ 30, and have ≥5 total supporting reads. Shared high-confidence SVs were identified by intersecting filtered Manta and DELLY calls using BEDTools with a reciprocal overlap threshold of ≥50%. The resulting shared SV dataset was used for downstream chromosome-level summaries, SV/Mb normalization, and linear and circos-based visualization.

### 2.4. Variant Calling and Annotation

Variant discovery was performed using the Genome Analysis Toolkit (GATK) best-practices workflow. Variants were initially called for each sample using HaplotypeCaller in gVCF mode. Individual gVCF files were combined using GenomicsDBImport, followed by joint genotyping with GenotypeGVCFs to generate a cohort-level variant call set.

Variants were filtered to retain high-confidence calls based on quality metrics, including a minimum read depth threshold (DP ≥ 60) and quality score (QS ≥ 30). Only variants labeled as PASS were retained for downstream analyses. Functional annotation was performed using snpEff to predict variant consequences and categorize variants based on genomic context and predicted impact.

### 2.5. Genome-Wide Variant Characterization

Following variant calling and filtering, variants were classified by type, including single-nucleotide variants (SNVs), insertions (INS), and deletions (DEL). Functional consequences were grouped into biologically relevant categories, including synonymous, missense, splice-region, frameshift, and stop-gain variants. Genome-wide distribution of variants was assessed by calculating variant counts across chromosomes and evaluating patterns of variant density. These analyses were used to characterize the overall structure and genomic distribution of variation within the dataset.

### 2.6. Allele Frequency and Rarity Analysis

Allele frequencies were calculated across the control cohort (*n* = 40) using the jointly genotyped variant dataset. Minor allele frequency (MAF) values were determined based on alternate allele counts across control samples. Variants present in the diseased horse were extracted and classified according to genotype state (heterozygous or homozygous alternate). Variants absent from all control samples were defined as private variants.

To support rare disease variant prioritization, variants were stratified into rarity categories based on allele frequency within the control cohort, including absent in controls (private), rare (AF < 0.01), low-frequency (AF 0.01–0.05), and common (AF > 0.05). These categories were used to assess the distribution of variant frequencies in the diseased sample relative to the population background.

### 2.7. Candidate Variant Prioritization

A sequential filtering strategy was applied to identify candidate variants associated with the disease phenotype. Variants were first restricted to high-confidence calls (PASS). Variants were then limited to protein-coding and splice-associated regions. Functional annotations were used to retain variants predicted to have moderate or high impact on protein function.

Variants were further filtered based on allele frequency, retaining rare and private variants within the control cohort. The resulting candidate variants were classified according to predicted functional consequence, including missense, frameshift, stop-gain, splice-site, and in-frame insertion or deletion variants.

### 2.8. Pathway Enrichment Network

The enrichment analysis was performed using a gene list derived from variants of interest identified in the whole-genome sequencing datasets and only moderate-to-high-impact mutations were used. Gene Ontology (GO) enrichment analysis was performed in R (version 4.5.2) using the gprofiler2 package with *Equus caballus* specified as the reference. Functional enrichment analysis was conducted using g:Profiler. The gene list was compared the *Equus caballus* reference annotation database to identify significantly enriched GO terms across the Biological Process (GO:BP), Molecular Function (GO:MF), and Cellular Component (GO:CC) ontologies. Multiple testing corrections were applied using the Benjamini–Hochberg false discovery rate (FDR) method. Enriched GO terms were considered significant at FDR-adjusted *p*-values < 0.05.

To visualize functional relationships among enriched pathways, an enrichment map network was generated in R using the igraph, tidygraph, and ggraph packages. Nodes represented significantly enriched GO terms, with node size corresponding to the number of genes contributing to each term and node color representing enrichment significance (−log10 FDR). Edges were drawn between nodes when GO terms shared overlapping gene sets, with edge width proportional to the degree of gene overlap. Additionally, a bar plot of enriched GO terms was generated using ggplot2, where enrichment significance was represented by the −log10(FDR) transformation of the corrected *p*-values.

### 2.9. Scratch Wound Assay/Invasion Assay

Cells were seeded at 1.0 × 10^6^ cells per well and cultured at 37 °C in a 24-well plate until reaching 100% confluency. To generate a consistent migration gap, a 2-well culture insert in a µ-Dish of size 35 mm (ibidi, Gräfelfing, Germany) was positioned in the center of each well, creating two separate cell compartments with a defined, uniform gap between them which serves like a scratch wound assay but with improved reproducibility. Once a confluent monolayer was established, the insert was removed to initiate migration into the gap. Cells were maintained under standard culture conditions during imaging, and brightfield images were collected at 10× magnification every 1 h using a plate reader equipped with 5% CO_2_ and environmental control to continuously monitor cell movement and gap closure over a 48-h period in DMEM media with 10% FBS.

Transwell migration assays were performed using a 24-well format with inserts containing 6 µm pores. The upper surface of each insert membrane was coated with Matrigel (30 µL per insert) and incubated at 37 °C to allow polymerization and formation of a barrier layer. Two experimental conditions were evaluated. In the first condition, ELAT cells were seeded onto the Matrigel-coated inserts to assess baseline migration with 20 thousand cells seeded. In the second condition, ELAT cells were first established as a confluent cellular layer on the insert membrane to serve as an endothelial barrier. Subsequently, colorectal cancer cell lines (Caco-2, HT-29, SW480, and SW620) were seeded in the upper chamber above the ELAT layer; for each colorectal cancer cell line, there were 50 thousand cells seeded. Cells in both the upper and lower chambers were suspended in DMEM with 10% FBS to facilitate migration. Plates were incubated at 37 °C with 5% CO_2_ for 24 h. Following incubation, cells that migrated through the membrane and adhered to the underside of the insert, as well as cells present in the lower chamber, were collected by trypsinization for 10 min. Trypsin was neutralized with complete medium, and the recovered cells were quantified using a cell counter.

Generative artificial intelligence (GenAI) tools were used to assist with language editing and grammar correction. Specifically, Grammarly (Grammarly Inc., San Francisco, CA, USA) was used during manuscript preparation. The authors reviewed and edited all outputs and take full responsibility for the accuracy and integrity of the final content.

## 3. Results

Genomic DNA was obtained from lymph node samples of a 3-year-old Quarter Horse mare, with a history of progressive, ulcerative skin lesions beginning 2 years prior. A total of 1,154,425,133 paired-end reads were generated, 99.86% mapped to the EquCab3.0 reference genome. A total of 97.81% of reads were properly paired, with 0.05% singletons. Following duplicate-marking, the dataset achieved a mean genome coverage of 39.8× with 97.98% of bases covered at the ≥10× depth, 92.84% at ≥20×, and 89.41% at ≥30×. The per-base sequence quality remained above Q30, and the GC content was approximately 43%.

**Figure 1:**
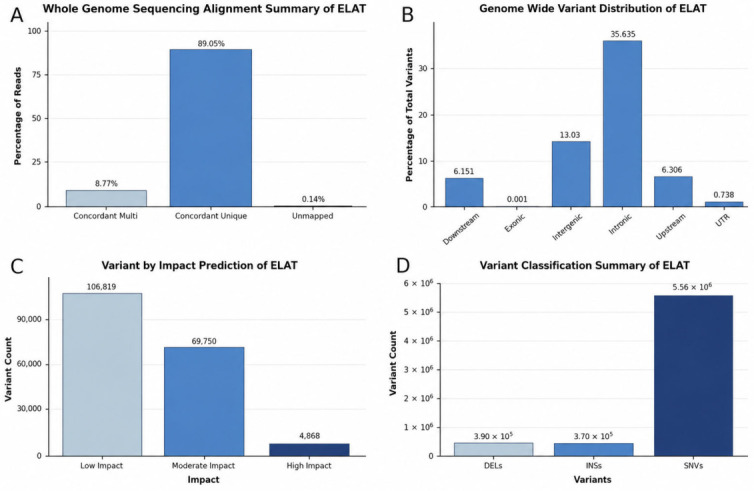
Overview of whole-genome sequencing alignment and variant characterization. (**A**) Alignment statistics for whole-genome sequencing reads, including concordant multi-mapped, concordant uniquely mapped, and unmapped reads. (**B**) Genome-wide distribution of variants across genomic regions, including downstream, intergenic, intronic, upstream, and untranslated regions. (**C**) Distribution of variants by predicted functional impact, including high-, moderate-, and low-impact categories. (**D**) Variant classification summary, including deletions (DELs), insertions (INSs), and single-nucleotide variants (SNVs).

Prior to the comparative analysis with the other 40 WGs, the variant-calling results showed a variant rate of 1 every 391 bases, totaling 6,343,906 variants processed after filter and non-variant. Of these, 5,576,463 were single-nucleotide variants (SNVs), 369,754 were insertions variants (INSs), and 397,689 were Deletions variants (DELs). A total of 4868 variants were classified as high-impact, 69,750 as moderate-impact, and 106,819 as low-impact based on the predicted functional consequences. Of the identified variants, 44.30% are missense, 0.35% are nonsense, and 55.35% were silent variants, with a missense–silent ratio of 0.80 (Figure 1).

### 3.1. Rarity Context Plots

**Figure 2:**
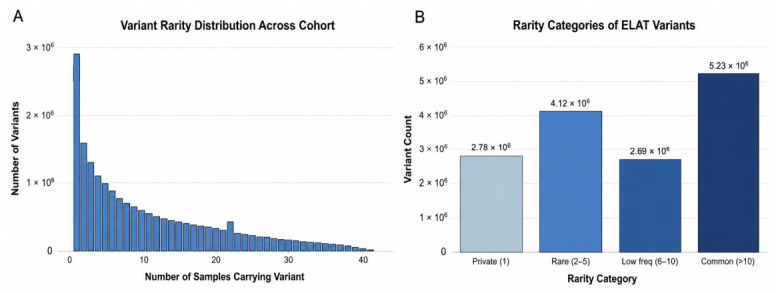
Distribution and classification of variant rarity across cohort. (**A**) Distribution of variants based on the number of samples in which each variant was observed. (**B**) Categorization of variants based on allele frequency, including private, rare, low-frequency, and common variants.

Variant rarity analysis demonstrated a strongly right skewed distribution across the cohort, with the majority of variants observed at low allele counts (Figure 2A). Most variants were present in only a small number of individuals, with a progressive decline in frequency as the number of samples carrying each variant increased. The stratification of variants carried by the ELAT sample showed representation across all rarity categories, including private, rare, low-frequency, and common variants (Figure 2B). A substantial proportion of variants were classified as private or rare within the cohort.

### 3.2. Functional Impact of Prioritized Variants

**Figure 3:**
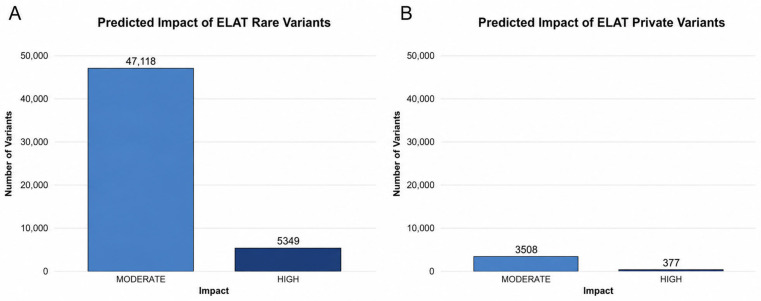
Predicted functional impact of private and rare variants. (**A**) Distribution of private variants by predicted functional impact, including high- and moderate-impact mutations. (**B**) Distribution of rare variants by predicted functional impact, including high- and moderate-impact mutations.

Among the prioritized variants, moderate-impact variants comprised the majority in both private and rare variant categories (Figure 3). Within private variants, 3508 were classified as moderate-impact and 377 as high-impact (Figure 3A). Similarly, among rare variants in the population, 47,118 were moderate-impact and 5350 were high-impact (Figure 3B). The distribution of variant impact classes was comparable between the private and rare variant groups, with moderate-impact variants representing the predominant class in each.

**Figure 4:**
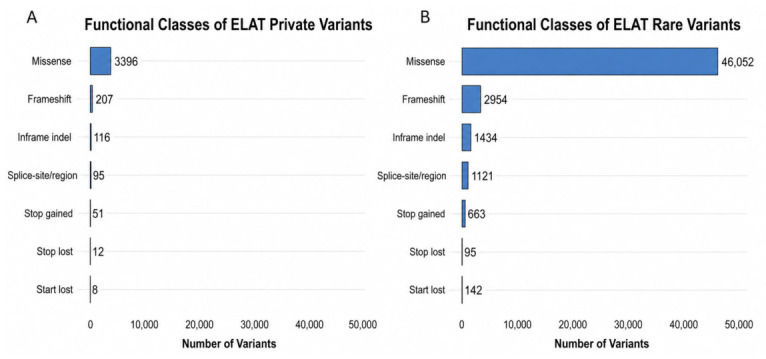
Functional classification of private and rare variants. (**A**) Bar plot showing the distribution of private variants across functional classes, including missense, frameshift, in-frame indels, splice-site/region, stop-gain, stop-loss, and start-loss variants. (**B**) Bar plot showing the distribution of rare variants across functional classes, including missense, frameshift, in-frame indels, splice-site/region, stop-gain, stop-loss, and start-loss variants. In both panels, bars represent variant counts within each functional class, and bar length is scaled according to the number of variants.

The functional classification of the prioritized variants revealed that missense variants were the most abundant class in both the private and rare variant groups (Figure 4). Within private variants, 3394 were classified as missense, followed by 207 frameshift variants, 114 in-frame insertions and deletions, 91 splice-site and region variants, 53 stop-gain variants, 18 stop-loss variants, and 8 start-loss variants (Figure 4A). A similar distribution was observed among rare variants, with 46,052 missense variants representing the largest category, followed by 2954 frameshift variants, 1434 in-frame insertions/deletions, 1122 splice-site/region variants, 663 stop-gain variants, 95 stop-loss variants, and 142 start-loss variants (Figure 4B). Across both variant groups, missense variants comprised the predominant functional class, while frameshift, splice-site region, and other coding disruptive variants were present at a lower frequency.

### 3.3. Gene-Level Prioritization and Burden Analysis

**Figure 5:**
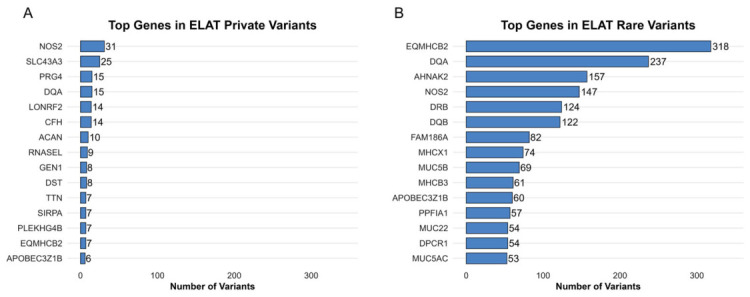
Genes containing the highest number of private and rare variants. (**A**) Bar plot of genes containing the highest number of private variants. (**B**) Bar plot of genes containing the highest number of rare variants. In both panels, bars represent individual genes and are scaled according to the number of variants identified in each gene.

The gene-level analysis of the prioritized variants identified multiple genes with elevated variant counts in both the private and rare variant groups (Figure 5). Within private variants, the highest variant burdens were observed in *NOS2* (31 variants) and *SLC43A3* (25 variants), followed by *PRG4* and *DQA* (15 variants each), *LONRF2* and *CFH* (14 variants each), and additional genes including *ACAN*, *RNASEL*, *GEN1*, and *DST* (Figure 5A).

Among rare variants, the greatest variant counts were observed in *EQMHCB2* (2406 variants), *TTN* (2350 variants), and *OBSCN* (2040 variants), followed by *PPFIA1* (1093 variants), *MACF1* (1091 variants), and *NOS2* (921 variants). Additional genes with elevated variant counts included *DST*, *SYNE1*, *DQA*, and *MDC1* (Figure 5B).

Across both variant groups, multiple genes exhibited recurrent variant enrichment, including *NOS2*, *TTN*, *DST*, and *DQA*, which were present among the top ranked genes in both the private and rare variant analyses.

**Figure 6:**
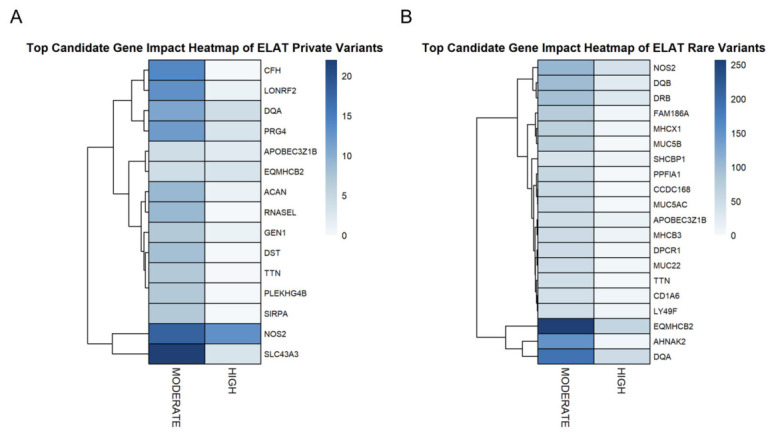
Heatmaps of predicted functional impact for candidate genes containing private and rare variants. (**A**) Heatmap of candidate genes containing private variants, displaying the distribution of moderate- and high-impact variants across genes. (**B**) Heatmap of candidate genes containing rare variants, displaying the distribution of moderate- and high-impact variants across genes. In both panels, color intensity represents the number of variants within each impact category for each gene, with darker colors indicating higher numbers of variants.

A heatmap of the top candidate genes demonstrated variation in the distribution of moderate- and high-impact variants across prioritized genes (Figure 6). In the private variant set, genes such as *NOS2* and *SLC43A3* exhibited the highest counts of moderate-impact variants, while high-impact variants were present at lower frequencies across all genes (Figure 6A).

In the rare variant set, elevated moderate-impact variant counts were observed in genes including *EQMHCB2*, *TTN*, and *OBSCN*, with comparatively lower counts of high-impact variants across the same gene set (Figure 6B). The clustering of genes based on their variant impact profiles revealed the grouping of genes with similar distributions of moderate- and high-impact variants within each dataset.

### 3.4. Genomic Distribution of Variants

**Figure 7:**
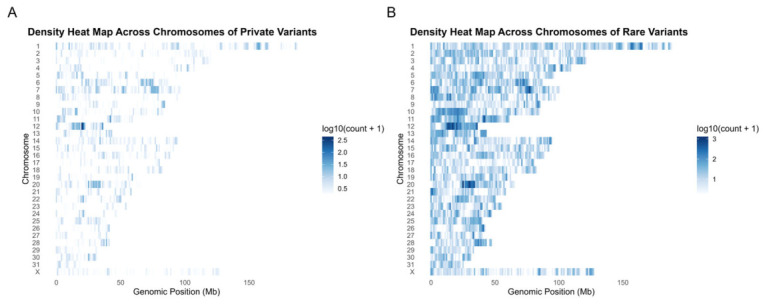
Chromosomal density heatmaps of private and rare variants. (**A**) Heatmap showing the distribution of private variants across chromosomes by genomic position. (**B**) Heatmap showing the distribution of rare variants across chromosomes by genomic position. In both panels, rows represent chromosomes and columns represent genomic position. Color intensity represents variant density as log_10_(count + 1), where counts are scaled to improve visualization and rows with zero counts are retained, with darker colors indicating higher variant density.

The heatmap visualization of the variant density across chromosomes demonstrated the nonuniform distribution of variants along both the private and rare variant datasets (Figure 7A,B). The variants were distributed across all chromosomes, with the signal intensity varying along the chromosomal positions, reflecting the differences in the local variant density. In the private variant dataset (Figure 7A), the variant signals were dispersed across chromosomes with intermittent regions of increased density, particularly in chromosome 12. These regions appeared as localized clusters of higher intensity interspersed among broader regions of lower variant representation, with variability observed both within and between chromosomes.

In the rare variant dataset (Figure 7B), a more continuous distribution of variant density was observed across chromosomes, with a greater number of genomic positions exhibiting detectable variant signals. Regions of elevated density were present across multiple chromosomes, forming extended segments of higher signal intensity relative to the surrounding regions. Differences in the signal intensity and continuity were evident between the private and rare variant sets, reflecting the variation in the distribution of variant counts across genomic positions.

**Figure 8:**
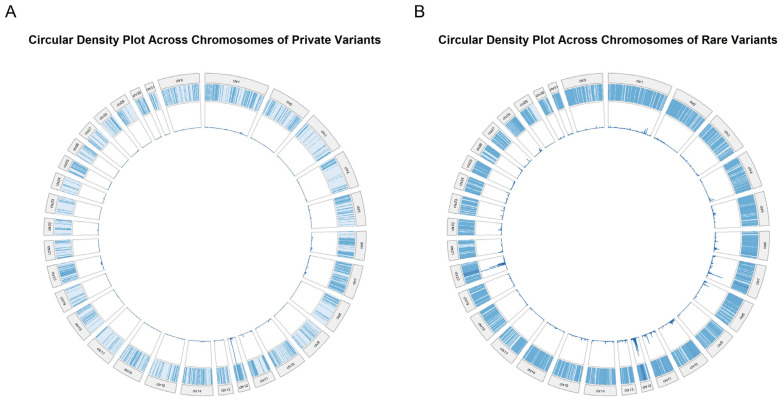
Circular density plots of private and rare variants across horse chromosomes. (**A**) Circular plot showing the distribution of private variants across horse chromosomes. (**B**) Circular plot showing the distribution of rare variants across horse chromosomes. In both panels, each segment represents a chromosome and genomic position along the chromosome, with radial bars indicating variant density. Bar height and color intensity reflect variant density, with darker and taller bars indicating higher variant density.

The circular genome visualization demonstrated the distribution of the variant density across all equine chromosomes in both the private and rare variant datasets (Figure 8). Variants were detected across all autosomes and the X chromosome, with the signal present throughout the genome. The radial density tracks indicated localized peaks in variant counts across multiple chromosomal segments, with variability observed between chromosomes. Across both datasets, the circular plots demonstrated the genome-wide distribution of variants with differences in the signal intensity and continuity between the private and rare variant groups. The chromosome-specific variation in density patterns was observed, with multiple regions exhibiting localized increases in variant counts.

### 3.5. Functional Enrichment and Network Analysis

**Figure 9:**
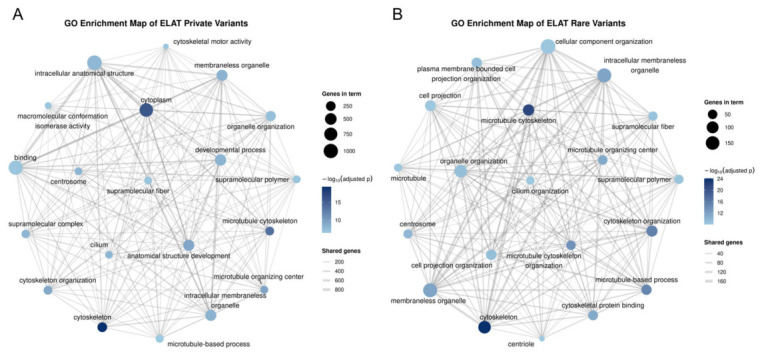
Gene Ontology (GO) enrichment maps of private and rare variants. (**A**) GO enrichment network of genes associated with private variants. (**B**) GO enrichment network of genes associated with rare variants. In both panels, nodes represent enriched GO terms, with node size corresponding to the number of genes in each term and node color representing enrichment significance as −log_10_ adjusted *p*-values, with darker colors indicating higher −log_10_ adjusted *p*-values. Edges indicate shared genes between GO terms, with edge thickness reflecting the number of shared genes. Nodes are labeled to reflect their corresponding Gene Ontology GO terms.

The Gene Ontology enrichment analysis of genes harboring moderate- and high-impact variants revealed significant enrichment across multiple cellular structural and cytoskeletal pathways in both the private and rare variant datasets (Figure 9). In the private variant dataset (Figure 9A), enriched terms included cytoskeleton, cytoplasm, and cytoskeleton organization, along with categories such as supramolecular complex, cytoskeletal protein binding, and microtubule-associated structures.

In the rare variant dataset (Figure 9B), enrichment was observed across a broader set of cytoskeletal and microtubule-related pathways. The most prominent terms included cytoskeleton, microtubule cytoskeleton, cytoskeleton organization, and microtubule-based processes, which showed higher enrichment values and larger gene counts relative to the private variant dataset. Additional enriched categories included the microtubule organizing center, cell projection organization, intracellular organelle organization, and supramolecular fiber structures.

The network visualization of enriched GO terms revealed interconnected clusters in both datasets, with substantial gene overlap between pathways (Figure 9). In the private variant network, terms formed a connected but more compact structure with fewer high-degree nodes. In contrast, the rare variant network exhibited a more extensive and densely connected topology, with central nodes such as cytoskeleton and microtubule-associated processes linking multiple functional categories.

**Figure 10:**
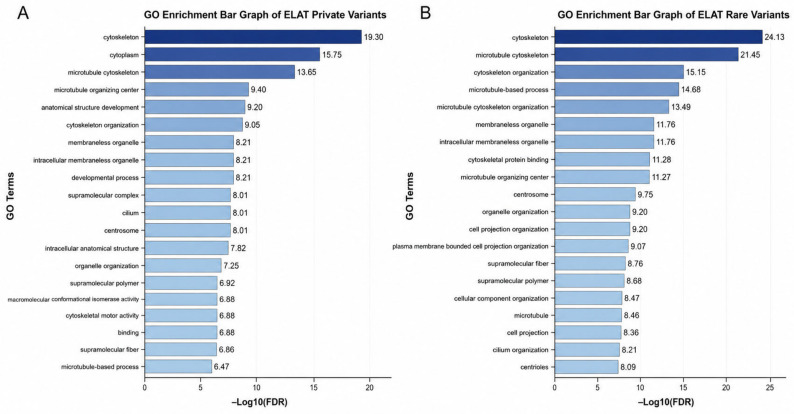
Gene Ontology (GO) enrichment bar plots of private and rare variants. (**A**) Bar plot of enriched GO terms associated with private variants. (**B**) Bar plot of enriched GO terms associated with rare variants. In both panels, bars represent GO terms and are ordered by enrichment significance expressed as −log_10_ adjusted *p*-values, whereby smaller *p*-values are converted into larger values such that higher values indicate greater statistical significance. Bar color intensity reflects enrichment significance, with darker shades corresponding to higher −log_10_ adjusted *p*-values. Each bar is labeled with its corresponding Gene Ontology (GO) term, and bar length is scaled according to enrichment significance.

The Gene Ontology enrichment analysis identified significant enrichment across multiple cytoskeletal and structural pathways in both the private and rare variant-associated gene sets (Figure 10). In the private variant dataset (Figure 10A), the most enriched terms included cytoskeleton and microtubule cytoskeleton, followed by cytoplasm and cytoskeleton organization. Additional enriched categories included supramolecular complex, cytoskeletal motor activity, supramolecular polymer, and supramolecular fiber. Terms associated with protein conformation and isomerase activity, including polypeptide conformation or assembly isomerase activity and macromolecular conformation isomerase activity, were also represented. Further enrichment was observed in cytoskeletal protein binding, polymeric cytoskeletal fiber, membraneless organelle, intracellular membraneless organelle, and centrosome. The enrichment values in this dataset were moderate across terms, with cytoskeleton showing the highest −log10(FDR).

In the rare variant dataset (Figure 10B), a broader range of cytoskeletal and microtubule-associated terms exhibited higher enrichment values. The most enriched terms included cytoskeleton and microtubule cytoskeleton, followed by cytoskeleton organization, microtubule-based process, and microtubule cytoskeleton organization. Additional enriched categories included membraneless organelle, intracellular membraneless organelle, cytoskeletal protein binding, microtubule organizing center, and centrosome. Terms related to the cellular structure and organization, including cell projection organization, organelle organization, plasma-membrane-bounded cell projection organization, and cellular component organization, were also enriched. Additional categories such as microtubule, cell projection, cilium organization, and centriole were represented. Enrichment values in the rare variant dataset were consistently higher across terms, with several categories demonstrating elevated −log10(FDR) compared to the private variant dataset. Across both datasets, enriched terms spanned multiple Gene Ontology domains, including biological process, cellular components, and molecular function. 

**Figure 11:**
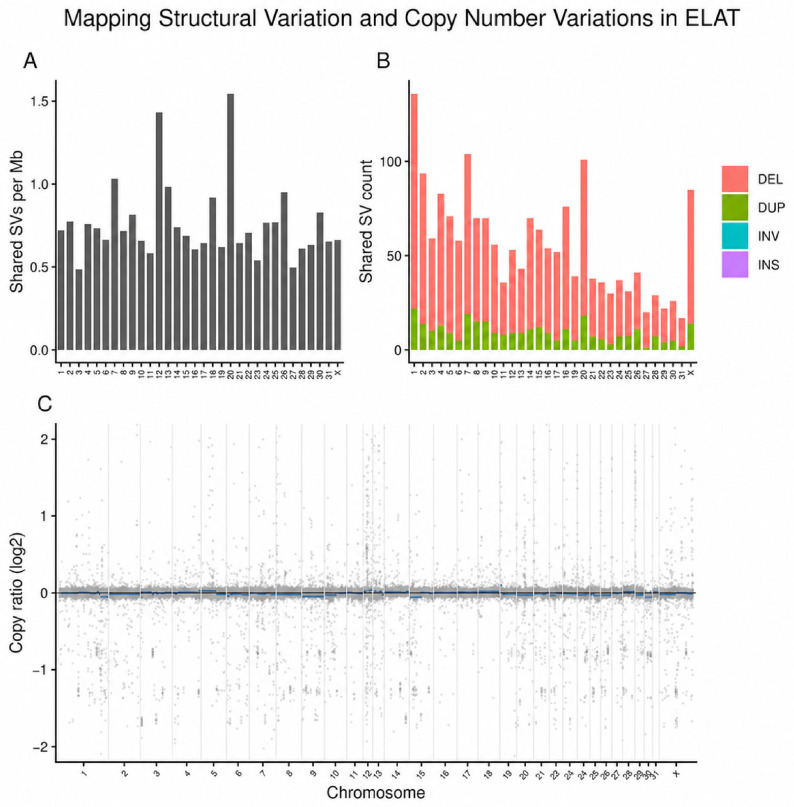
Mapping structural variation and copy number variation in ELAT. (**A**) Chromosome-level distribution of structural variants (SVs) between Manta and DELLY, normalized as SVs per megabase (SV/Mb). (**B**) Total shared SV counts per chromosome stratified by variant type, including deletions (DELs), duplications (DUPs), inversions (INVs), and insertions (INSs). (**C**) Genome-wide copy number variation profile generated using CNVkit: each point represents a 50 kb bin with log2 copy ratio values plotted across chromosomes.

High-confidence structural variants were identified across the primary chromosomes of the ELAT sample. When normalized by the chromosome length, the shared SV density was generally consistent across chromosomes, with most chromosomes showing comparable SVs per megabase (Figure 11A). When shared SVs were separated by variant type, deletions represented the most frequent SV class across the genome, followed by duplications. Inversions and insertions were detected at lower frequencies (Figure 11B). CNVkit analysis showed that most genomic bins clustered around a log2 copy ratio of zero, consistent with a predominantly diploid copy number profile. No chromosome-wide gains or losses were observed across the primary chromosomes (Figure 11C).

**Figure 12:**
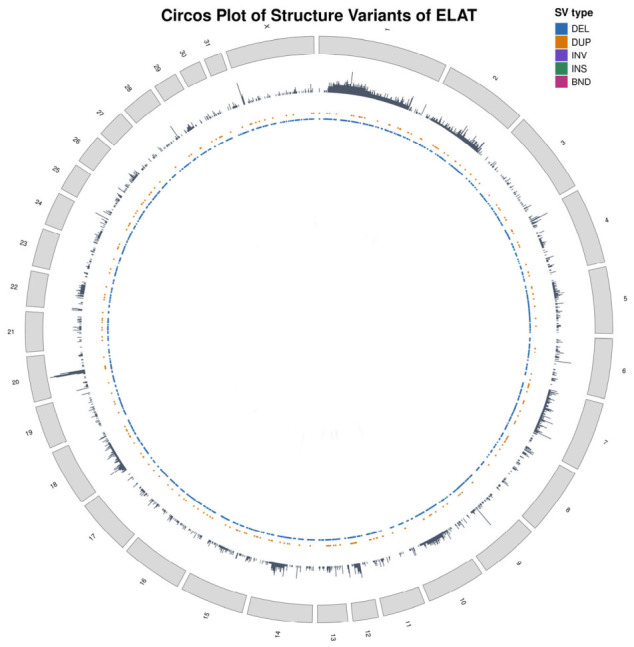
Circos plot of structure variants of ELAT. Circos represents structural variants across primary chromosomes. The outer ideogram shows chromosome position, while inner tracks summarize SV distribution and variant class, including deletions.

Figure 12 shows the genome-wide distribution of structural variants across the ELAT primary chromosomes using a circos-based visualization. Structural variants were detected across multiple chromosomes rather than being restricted to one chromosome or a small number of regions. The outer SV density track shows variation in the SV signal among chromosomes, with larger chromosomes generally containing more events.

The inner tracks show that deletions and duplications were the most frequent SV classes, while inversions, insertions, and breakend (BND) events were less common and did not appear concentrated in one region. Overall, the circos plot shows a broadly distributed SV pattern across the ELAT genome, without a dominant chromosome-wide structural abnormality.

**Figure 13:**
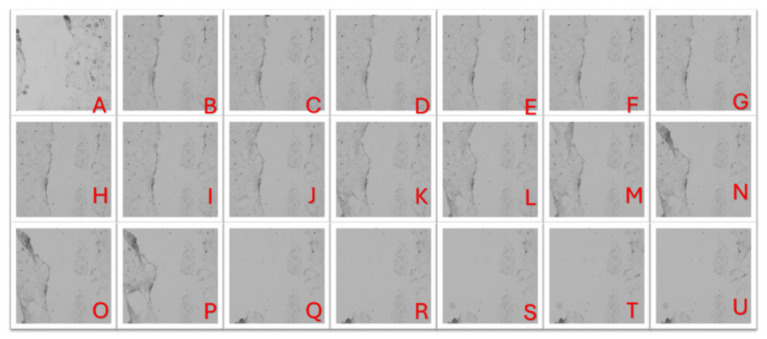
Representative still frames from the scratch wound time-lapse video (Supplementary S5) captured at 2-h intervals. Panels (**A**–**U**) show the wound region over time, showing a persistent cell-free gap with minimal edge advancement and no evident wound closure during the imaging period.

Time-lapse imaging (Figure 13, Supplementary S5) shows that the scratch injury produced a well-defined, cell-free gap between cell regions. The wound margins remained clearly defined throughout the recording, with little to no inward migration from the edges. Rather than forming a cohesive leading front, the monolayer along the boundary became discontinuous as cells retracted and aggregated into dense clumps, resulting in an uneven, patchy coverage at the wound. Small clusters of elongated cells were visible within the field of view, and the debris-consistent procedure persisted over time. Overall, the wound did not show evidence of closure at any point during the experiment.

**Figure 14:**
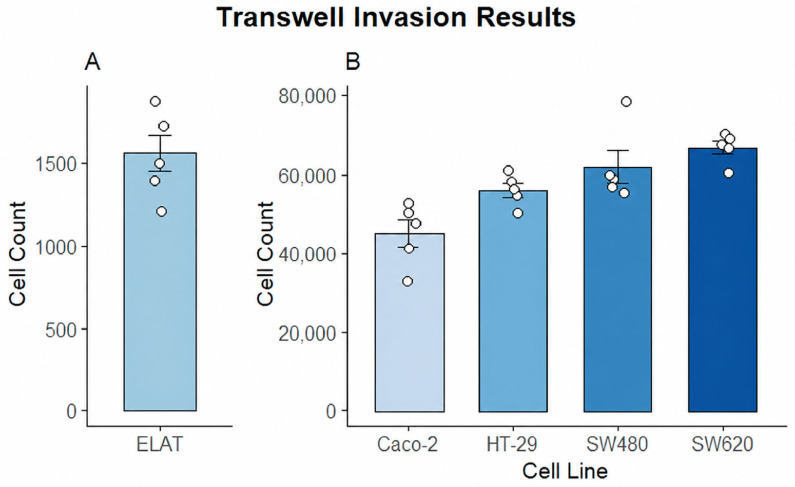
Transwell invasion assay. (**A**) MEED cells seeded onto Matrigel-coated Transwell inserts: cells that migrated into the lower chamber were collected and quantified. (**B**) Colorectal cells seeded above an ELAT monolayer on Matrigel-coated Transwell.

ELAT cells were seeded at 20,000 cells and analyzed separately from the colorectal cancer cell lines (Figure 14). After 24 h, ELAT showed a mean recovered cell count of 1558 cells, with individual replicate values ranging from approximately 1200 to 1800 cells. For the colorectal cancer cell lines, cells were seeded at 50,000 cells under the co-culture Transwell condition. Caco-2 showed the lowest recovered cell count among the colorectal cancer cell lines, with a mean of 44,831 cells and replicate values ranging from 32,840 to 52,500 cells. HT-29 showed a higher mean recovered cell count of 55,999 cells, with replicate values ranging from 50,124 to 61,025 cells. SW480 showed a mean recovered cell count of 61,860 cells, with values ranging from 55,421 to 78,410 cells. SW620 showed the highest recovered cell count, with a mean of 66,679 cells and replicate values ranging from 60,210 to 70,245 cells.

## 4. Discussion

Multisystemic eosinophilic epitheliotropic disease (MEED) is a poorly understood condition characterized by chronic eosinophilic inflammation, epithelial disruption, and progressive multi-organ involvement [3,5,6,7]. Despite its clinical severity, the underlying genetic and molecular mechanisms driving disease onset and progression remain largely undefined, with the current understanding primarily centered around inflammation and immune-mediated processes [3,6,7,8]. However, the extent of the epithelial damage and tissue remodeling observed in MEED suggests that additional factors beyond inflammatory and immune dysregulation alone may contribute to the disease pathogenesis.

In this study, whole-genome sequencing was used to explore the genetic architecture of MEED, providing a framework for identifying candidate variants potentially associated with the disease. The sequencing of the affected mare produced high-quality genomic data with strong coverage and mapping metrics, supporting the reliability of downstream variant identification. The overall variant burden and distribution were consistent with equine genome expectations, while the enrichment of moderate- and high-impact variants provided a focused subset for prioritization. The variant landscape was dominated by low-frequency alleles, with a substantial proportion of variants classified as private or rare within the whole cohort. This distribution supports the premise that possible disease-associated variants in this case are more likely to reside within these low-frequency categories rather than among common population mutations. Within the rare and private variant space, moderate-impact variants that are predominantly missense changes (Figure 4) represented the largest functional class, though some of the listed high-impact variants could still play a role. Collectively, this pattern supports a model in which the phenotype may arise from the combined effects of multiple variants with modest functional consequences, consistent with a complex or multifactorial disease process.

Genome-wide analyses of private variants unique to the affected horse demonstrated that variants were distributed across all chromosomes, with localized regions of increased variant density, particularly on chromosomes 1, 6, 7, 12, and 20 (Figure 7 and Figure 8). However, while these chromosomes have an increased variant density, it does not necessarily correspond to the functional impact, as several top candidate genes such as *NOS2* and *SLC43A3* (Figure 6) are located on chromosomes 11 and 3 [10]. This suggests that disease-related variation may arise from specific high- and moderate-impact genes rather than the overall variant burden within a given chromosome. Chromosome 12 exhibited the highest variant burden in our WGS analysis, this chromosome contains genes involved in the cellular architecture, nuclear-cytoskeletal organization, and responses to mechanical stress, all of which are critical for maintaining epithelial integrity [11,12]. The disruption of these pathways may compromise barrier function and increase susceptibility to the tissue damage observed in horses with MEED, although chromosome 12 has been listed as a commonly affected area in clinically normal horses and chromosome variant burden should not be directly correlated with clinical impact, so this result should be interpreted with care [12,13].

Gene-level analyses identified private recurrent variants in genes associated with immune regulation, epithelial integrity, and cytoskeletal organization. These findings align closely with the pathophysiology of MEED, which is characterized by chronic inflammation, epithelial infiltration, and tissue remodeling. An analysis of the top candidate genes helps to highlight possible key pathways involved in the clinical presentation, although no single gene fully explains the observed phenotype. Variants in *NOS2*, a key regulator of nitric oxide production, suggest the potential dysregulation of inflammatory signaling pathways [14,15]. Nitric oxide plays a central role in immune responses and tissue injury; altered *NOS2* activity may contribute to the sustained inflammatory environments and epithelial damage observed in MEED lesions [16,17]. Excess nitric oxide production has also been shown to disrupt epithelial barrier functions through the altering of tight junction proteins and increasing oxidative stress, leading to an enhanced epithelial permeability^18^. This may promote conditions that facilitate immune cell infiltration, including eosinophils, perpetuating localized tissue damage. 

In parallel, *SYNE1* is a critical component of the linker of the nucleoskeleton and cytoskeleton complex (LINC), pointing towards an impaired cellular structural integrity [19,20,21]. *SYNE1* is responsible for maintaining nucleus positioning and transmitting mechanical forces across the cell, which are essential for epithelial stability and resilience [19,22,23]. The disruption of *SYNE1*-mediated pathways may weaken the cytoskeleton organization and reduce the ability of epithelial cells to withstand mechanical and inflammatory stress [19,21,24]. While *NOS2* and *SYNE1* highlight the key pathways related to nitric oxide signaling and cellular structural integrity, they only represent a part of a broader network of affected genes contributing to the clinical presentation [15,18,25,26,27]. Another top gene candidate, *SLC43A3*, a metabolic transport gene, encodes a nucleobase transporter *ENBT1* involved in nucleotide salvage pathways, which are essential for cellular metabolism and epithelial turnover [28,29]. In epithelial tissues, the disruption of these pathways could lead to a weakened barrier function, increase the permeability, and impair the regenerative capacity. This is particularly relevant in MEED, where epithelial surfaces are a primary site of pathology and exhibit progressive ulceration and structural breakdown.

The presence of variants in major histocompatibility complex-related genes, including *DQA* and *EQMHCB2*, further signifies the altered antigen presentation and immune activation. *DQA* encodes a component of the MHC class II molecule responsible for presenting extracellular antigens to CD4 T cells, while *EQMHCB2* is an MHC class I with the broader equine MHC region and contributes to antigen processing and immune recognition [25,26,27,28]. MEED is thought to involve aberrant immune responses with prominent eosinophilic infiltration, and the disruption of antigen presentation pathways may contribute to an inappropriate or sustained immune activation against epithelial tissues. This supports a model in which immune dysregulation could be a central contributing factor to the broader disease’s progression. 

The GO enrichment results help provide context for the patterns observed in both the heatmap and gene-level analyses. The most significantly enriched pathways were related to the cytoskeleton, microtubules, actin organization, and overall cellular structure, which aligns with the genes identified in the private variant group. Genes such as *TTN* and *OBSCN*, which are important for the sarcomere structure and muscle organization, along with *MACF1* and *DST*, which are involved in cytoskeletal linkage and actin–microtubule interactions, support the biological relevance of these enriched pathways [29,30,31,32,33]. These pathways are important for maintaining the cell shape, intracellular transport, and epithelial integrity, which are all critical in tissues affected in equine MEED. The connectivity seen in the enrichment network also suggests that many of these genes are functionally related, further supporting the idea that multiple moderate-impact variants across related pathways may be working together to cause disease, rather than a single major mutation. In the context of MEED, the disruption of cytoskeletal and actin-related pathways could have important effects on the epithelial barrier function and tissue stability [34,35,36]. Epithelial cells depend on an intact cytoskeleton to maintain tight junctions and to withstand both mechanical and inflammatory stress [32,33,34]. Because of this, alterations in these pathways may contribute to a decreased epithelial integrity and greater susceptibility to inflammation, which fits with clinical findings such as ulceration, scaling, and mucosal involvement.

Consistent with these genomic observations, exploratory functional assays were used to assess ELAT cell behavior. The scratch wound assay suggested a limited wound-closure capacity following mechanical disruption (Figure 12, Supplementary S5). Throughout the imaging period, ELAT monolayers maintained a persistent cell-free gap with minimal wound-edge advancement. Cells at the wound margin did not demonstrate clear directional migration, and there was no observable increase in cell density within the wound area. Instead, cells appeared to aggregate along the wound edge, which was associated with an apparent widening of the gap over time. These findings indicate that, under the conditions tested, ELAT cells did not exhibit the coordinated migratory or proliferative behavior typically associated with wound closure. In established epithelial and endothelial models, wound repair involves a transition to a motile phenotype characterized by cell polarization, cytoskeletal reorganization, focal adhesion turnover, and junctional remodeling [37]. The limited response observed in ELAT cells may suggest the reduced activation of one or more of these processes.

Invasion assays provided additional functional context. When assessed independently, ELAT cells demonstrated a low invasive capacity, with approximately 12% of seeded cells traversing the Matrigel-coated membrane over 24 h. In contrast, in a co-culture system where colorectal cancer cells were placed above an ELAT monolayer, a substantial number of cells were recovered in the lower chamber. Due to the high proliferative capacity of colorectal cells, this increase likely reflects a combination of invasion and proliferation during the assay period. These findings suggest that, under the conditions tested, the ELAT monolayer has a limited barrier function.

Structural variant and copy number analyses further support a genome that is largely stable at the chromosomal level. Copy number profiling showed no evidence of broad chromosomal gains or losses, while structural variants were distributed across the genome without a single chromosome demonstrating dominant enrichment after normalization. Although structural variants were detected across multiple chromosomes, their distribution did not indicate large-scale genomic rearrangements or chromosomal instability. Breakend (BND) events, representing potential intrachromosomal rearrangements, were present but occurred at a low frequency and did not form recurrent patterns.

While MEED is primarily described as an immune-mediated and inflammatory disease, these results suggest that additional genetic factors may be involved [3,5,6,7]. In particular, the enrichment of genes related to muscle structure, cytoskeletal organization, and actin dynamics raises the possibility of previously unrecognized structural or musculoskeletal components of the disease. However, it is important to note that tissue injury and inflammation can activate coordinated cellular responses, including cytoskeletal remodeling and epithelial repair, which may lead to the apparent enrichment of these pathways even when they are secondary to the underlying disease process. Therefore, while the pathways identified are clearly enriched and biologically relevant, further work is needed to determine whether these variants contribute directly to the disease pathology or whether the observed pathway enrichment reflects the secondary effects of chronic inflammation.

The primary limitation is the inclusion of only a single affected individual, which makes it difficult to distinguish potentially causative variants from background rare genetic variation. This challenge is further compounded by the rarity of multisystemic eosinophilic epitheliotropic disease (MEED), as the limited number of reported cases makes the collection of additional samples difficult, if not impossible. Additionally, genomic DNA was obtained from a cell culture derived from the affected individual, which introduces another source of potential bias. Cell lines are known to accumulate genetic mutations and chromosomal rearrangements over successive passages, and, therefore, some identified variants may reflect culture-associated changes rather than the in vivo genomic profile. However, whole-genome sequencing was conducted on passage 2, which lowers the chances of culture-associated changes. Given these limitations, all mechanistic interpretations presented here should be considered preliminary and hypothesis-generating, and further research is needed to confirm these findings. However, this exploratory genomic analysis provides a foundation for future studies aimed at better understanding the genetic basis of MEED.

Future studies should focus on functional validation through more experimental in vitro studies which are important in order to determine the true biological impact of candidate mutations identified in this study. In addition, future work could expand beyond protein-coding regions to include regulatory elements, which may play a significant role in disease development. Together, these efforts will help clarify the genetic basis and build a greater understanding of MEED.

## 5. Conclusions

In conclusion, this study suggests that MEED may involve a complex genetic architecture shaped by multiple rare or low-frequency variants rather than a single disease-causing mutation. Candidate variants associated with immune regulation, antigen presentation, cytoskeletal organization, extracellular matrix integrity, and epithelial barrier function provide a biologically plausible framework for the chronic eosinophilic inflammation, ulceration, and multisystemic progression characteristic of MEED. Given the rarity of MEED and the limited number of reported cases, these findings should be interpreted as hypothesis-generating and a framework to explore. Importantly, functional assays, including cell-based migration and invasion studies, provide preliminary biological support for the genomic findings by demonstrating altered epithelial behavior consistent with impaired barrier function and tissue remodeling. Together, these integrated approaches establish a foundation for future studies aimed at validating candidate variants and further defining the molecular mechanisms underlying MEED development.

## Data Availability

The whole-genome sequencing data of the MEED horse sample is available in the National Center for Biotechnology Information (NCBI) Sequence Read Archive under BioProject accession PRJNA1447573. Publicly available control datasets used for comparative analysis were obtained from the National Center for Biotechnology Information (NCBI) Sequence Read Archive under BioProject accession PRJNA993255. All bioinformatics analyses were conducted using established and publicly available tools, including FastQC, BWA-MEM, SAMtools, Picard, GATK, bcftools, and snpEff, along with custom scripts for variant filtering, prioritization, and visualization. Custom analysis scripts used for data processing, statistical analysis, and figure generation are available from the corresponding author upon request.

## Supplementary Materials

The following supporting information can be downloaded at: https://www.mdpi.com/article/10.3390/ani16101560/s1, Rare GO pathway Excel sheet (S1), Private GO pathway Excel sheet (S2), Private variant Excel sheet (S3), Rare variant Excel sheet (S4), Scratch Assay Movie (S5), PCA Graph of MEED Compared to Quarter Horse and Thoroughbred (S6), and Chromosome-Level Depth Profile (S7).
