# Supplementary figures and images for: Genomics in Equine MEED: Whole-Genome Sequencing and Target Mutation Identification
Source: Animals (Basel). 2026 May 21;16(10):1560. doi: 10.3390/ani16101560 (PMC13203250; doi:10.3390/ani16101560)

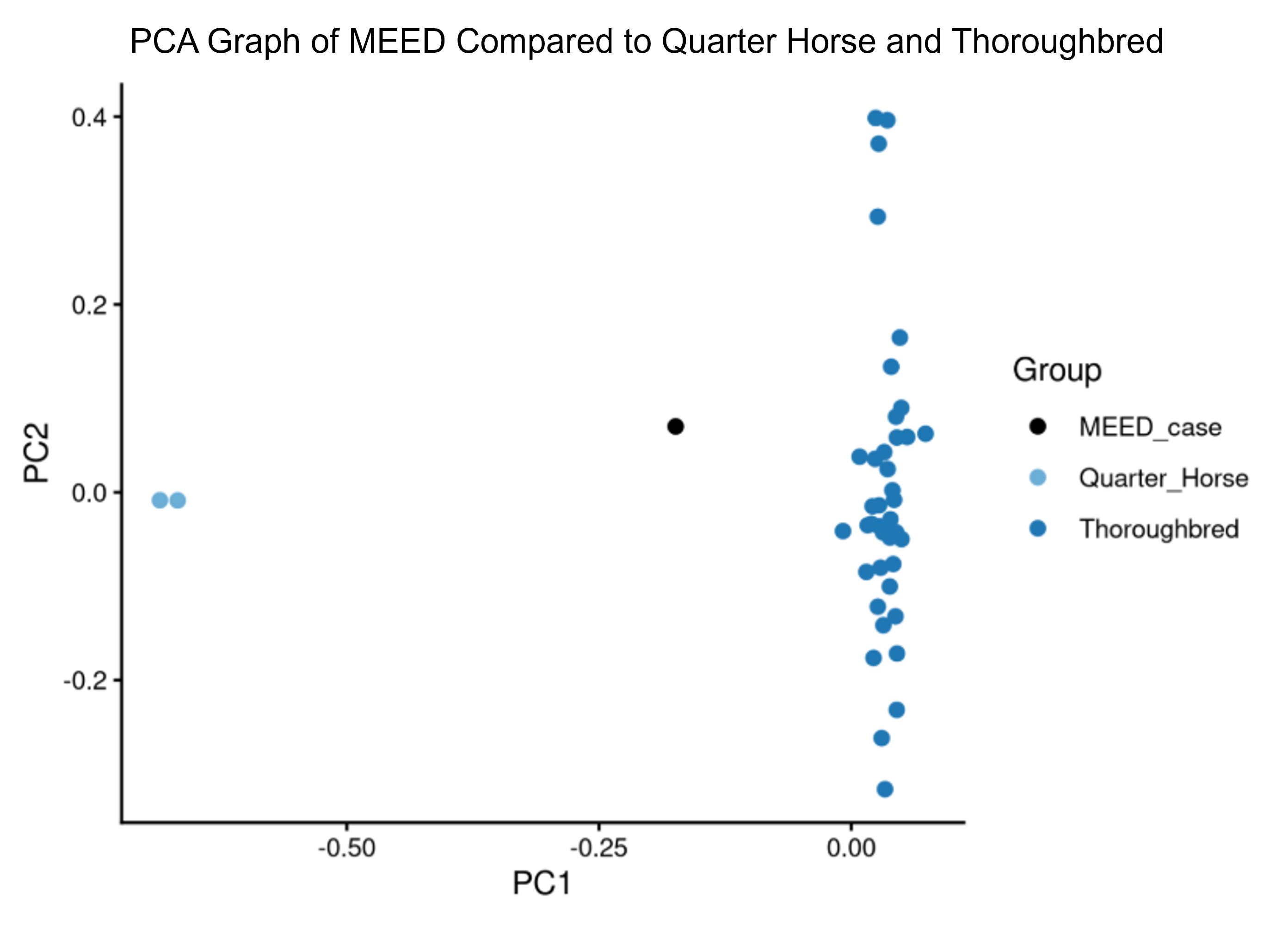

Supplement: Supplementary file 1 [file animals-16-01560-s001.zip › Supplemental S6.tif]

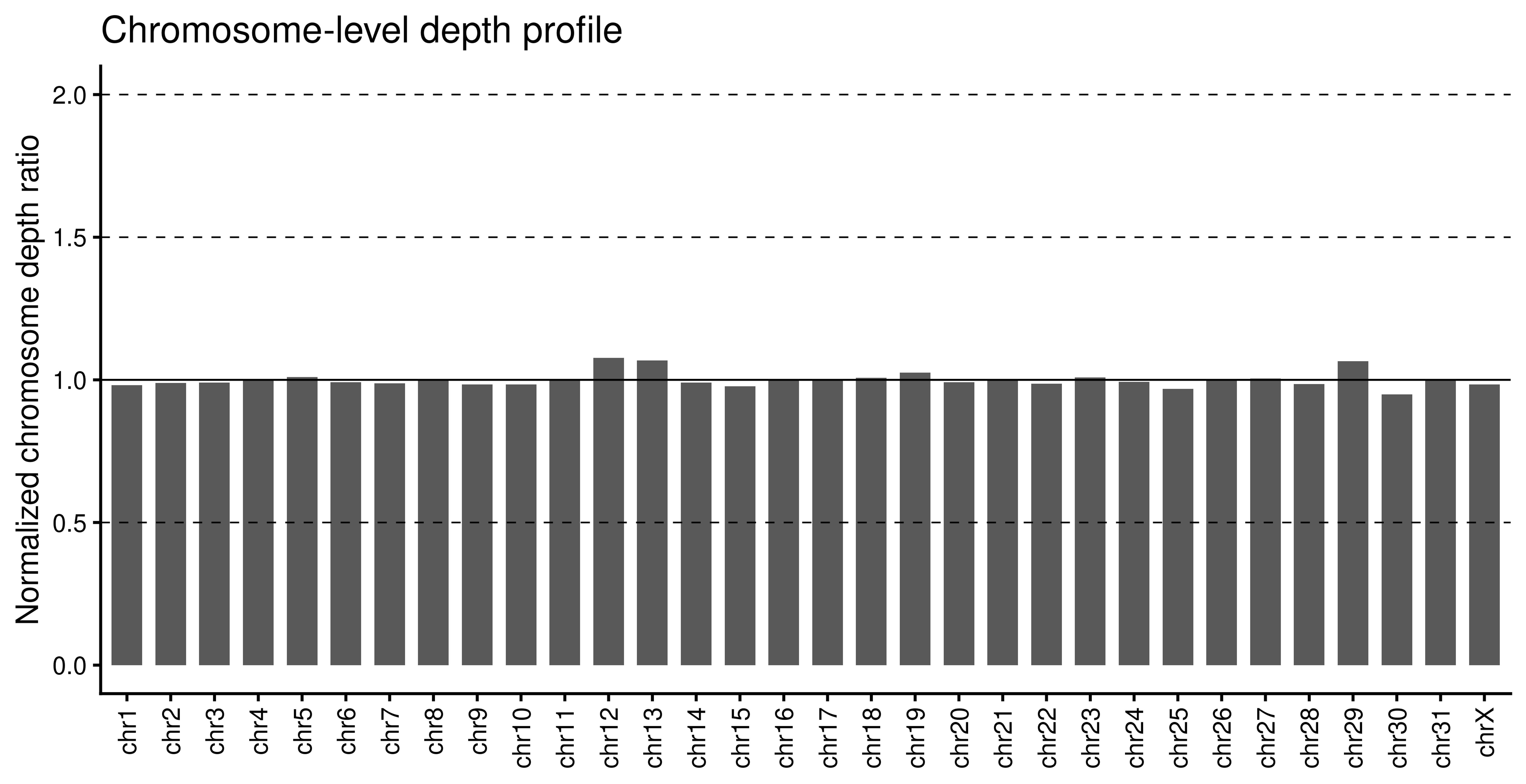

Supplement: Supplementary file 1 [file animals-16-01560-s001.zip › Supplemental S7.tif]
